# Distinct Bacterial Communities in Wet and Dry Seasons During a Seasonal Water Level Fluctuation in the Largest Freshwater Lake (Poyang Lake) in China

**DOI:** 10.3389/fmicb.2019.01167

**Published:** 2019-05-21

**Authors:** Ze Ren, Xiaodong Qu, Min Zhang, Yang Yu, Wenqi Peng

**Affiliations:** ^1^State Key Laboratory of Simulation and Regulation of Water Cycle in River Basin, China Institute of Water Resources and Hydropower Research, Beijing, China; ^2^Division of Biological Sciences, University of Montana, Missoula, MT, United States; ^3^Department of Water Environment, China Institute of Water Resources and Hydropower Research, Beijing, China

**Keywords:** 16S rRNA, nutrient, Changjiang River, shallow lake, hydrological regime

## Abstract

Water level fluctuations (WLFs) are an inherent feature of lake ecosystems and have been regarded as a pervasive pressure on lacustrine ecosystems globally due to anthropogenic activities and climate change. However, the impacts of WLFs on lake microbial communities is one of our knowledge gaps. Here, we used the high-throughput 16S rRNA gene sequencing approach to investigate the taxonomic and functional dynamics of bacterial communities in wet-season and dry-season of Poyang Lake (PYL) in China. The results showed that dry-season was enriched in total nitrogen (TN), nitrate (NO_3_^-^), ammonia (NH_4_^+^), and soluble reactive phosphorus (SRP), while wet-season was enriched in dissolved organic carbon (DOC) and total phosphorus (TP). Bacterial communities were distinct taxonomically and functionally in dry-season and wet-season and the nutrients especially P variation had a significant contribution to the seasonal variation of bacterial communities. Moreover, bacterial communities responded differently to nutrient dynamics in different seasons. DOC, NO_3_^-^, and SRP had strong influences on bacterial communities in dry-season while only TP in wet-season. Alpha-diversity, functional redundancy, taxonomic dissimilarities, and taxa niche width were higher in dry-season, while functional dissimilarities were higher in wet-season, suggesting that the bacterial communities were more taxonomically sensitive in dry-season while more functionally sensitive in wet-season. Bacterial communities were more efficient in nutrients utilization in wet-season and might have different nitrogen removal mechanisms in different seasons. Bacterial communities in wet-season had significantly higher relative abundance of denitrification genes but lower anammox genes than in dry-season. This study enriched our knowledge of the impacts of WLFs and seasonal dynamics of lake ecosystems. Given the increasingly pervasive pressure of WLFs on lake ecosystems worldwide, our findings have important implications for conservation and management of lake ecosystems.

## Introduction

Lakes fluctuate intra- and inter-annually in response to seasonal and long-term hydrologic imbalance, respectively. As a natural hydrological regime, water level fluctuations (WLFs) are an inherent feature of lake ecosystems and dominant forces controlling the structure and function of lacustrine ecosystems (Leira and Cantonati, 2008; Wantzen et al., 2008; Evtimova and Donohue, 2016). Any significant changes of water level can impose consequential effects on lake physical, chemical, and biological properties (Coops et al., 2003; Hofmann et al., 2008). For shallow lakes, seasonal pulses of water are especially important and a slight water level change can be translated to a substantial change of lake surface area and water volume (Feng et al., 2012; Han et al., 2015). Therefore, WLFs might have an overriding effect on the health and integrity of some lake ecosystems (Leira and Cantonati, 2008). However, these natural hydrological regimes are increasingly impacted by anthropogenic activities on local and global scales through dam construction, water extraction, as well as on-going climate change (Poff and Zimmerman, 2010; Haddeland et al., 2014).

A great deal of research has been conducted to assess the impacts of excessive WLFs on shallow lakes. These studies demonstrated dramatic effects of WLFs on physical properties (e.g., lake morphometry, sedimentation, light penetration, temperature regime, and residence time) (Nowlin et al., 2004; Zohary and Ostrovsky, 2011; Li et al., 2015), chemical environments (e.g., water quality and nutrient distribution and release) (Dinka et al., 2004; Yao et al., 2015; Li et al., 2016; Hideo et al., 2017), and biological populations and communities (e.g., macrophytes, algae, zooplankton, invertebrates, and fish) of lake ecosystems (Coops and Hosper, 2002; Evtimova and Donohue, 2016). In the past decades, natural lakes around the world have been experiencing dramatic changes in their size, morphology, and ecology (Awange et al., 2008), such as the Great Lakes in the United States (Assel et al., 2004; Clites et al., 2014), Lake Chad and Lake Victoria in Africa (Awange et al., 2008; Gao et al., 2011), PYL and Dongting Lake in China (Feng et al., 2012; Yuan et al., 2015; Han et al., 2018), and Lake Tonle Sap in Cambodia (Hideo et al., 2017). As the global climate change and anthropogenic activities increases, extreme WLFs are more frequent and have been becoming one of the main threats impairing the ecological integrity and security of lake ecosystems globally.

Despite becoming one of the main pressures on lake ecosystems globally and gaining growing research interests, the impact of WLFs are still not fully understood (Leira and Cantonati, 2008; Wantzen et al., 2008; Evtimova and Donohue, 2016). One of the serious knowledge gaps is the effect of WLFs on microbial communities. Microbial communities are fundamental components in lake ecosystems encompassing tremendous diversity and play a pivotal role in driving biogeochemical processes, including carbon, nitrogen, and phosphorus cycles (Whitman et al., 1998; Newton et al., 2011; Huang et al., 2016; Peter and Sommaruga, 2016). According to previous research, WLFs had significant impacts on many key environmental properties of lakes (White et al., 2008). Changes in lake environments can significantly shift structure and function of microbial communities (Farjalla et al., 2006; Pablo Nino-Garcia et al., 2016; Peter and Sommaruga, 2016). Thus, elucidating the seasonal dynamics of microbial assemblages during WLFs is of great interest and importance to get insights into the ecological impacts of WLFs and biogeochemical properties of the Lake.

Poyang Lake is the largest freshwater lake in China. Located in one of the most frequently drought and flooded areas in China, PYL has been experiencing dramatic inter- and intra-annual fluctuations especially huge seasonal changes in lake area with the maximum inundation area more than 14 times the minimum inundation area (Hui et al., 2008; Feng et al., 2012; Xu et al., 2014). Significant hydrological changes have become an important problem of ecological security in PYL. The Three Gorges Dam (TGD) further modulates the water balance of PYL and aggravates this extreme WLF especially the seasonal dryness since its impoundment in 2003 (Feng et al., 2013; Ye et al., 2014; Zhang et al., 2014; Mei et al., 2015). Under the dual pressure of climate change and TGD, PYL has intensified extreme WLFs with an advanced and prolonged dry-season (Zhang et al., 2011, 2012; Guo et al., 2012).

Water level fluctuations have complex influences on lake ecosystems. However, our knowledge about the dynamic of bacterial communities experiencing excessive seasonal WLFs is scarce. Given the increasingly pervasive pressure of WLFs on lake ecosystems worldwide, in this study, we examined the microbial communities in wet-season and dry-season of PYL using high-throughput 16S rRNA gene sequencing and assessed the functional potentials in carbon (C-), nitrogen (N-), and phosphorus (P-) cycles. Our aim is to reveal the spatiotemporal patterns of bacterial community distribution and their potential functions in biogeochemical cycles during an extreme seasonal WLF.

## Materials and Methods

### Study Area and Sample Collection

**FIGURE 1:**
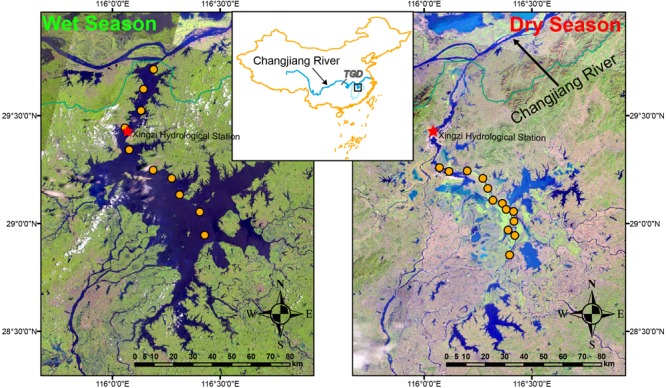
Study area and sample sites. Samples were collected from 10 and 13 sites in wet-season (July 2017) and dry-season (January 2017), respectively. TGD represents Three Gorges Dam, the world’s largest hydroelectric dam. The map was created in ArcGIS 14.0 (http://desktop.arcgis.com/en/arcmap/) using Landsat images download from USGS (https://earthexplorer.usgs.gov/). The images show the change of the lake area. The images were acquired in July and January 2017, respectively, corresponding to the sampling time.

Located in the lower reach of Changjiang River (Yangtze River), PYL is the largest freshwater lake in China as well as one of the two lakes that has retained its free connection to Changjiang River (Figure 1). There are five tributary rivers (Ganjiang, Fuhe, Xinjiang, Raohe, and Xiushui rivers) feeding PYL and the lake typically flows from south to north discharging to the Changjiang River finally. The annual runoff of PYL is 152.5 billion m^3^, accounting to 16.3% annual runoff of Changjiang River. PYL is a shallow seasonal lake and a typical water-carrying and throughput lake (Fang et al., 2011; Zhao et al., 2011). The lake region belongs to the East Asian Monsoon Region which leads to great seasonality in precipitation. Moreover, due to the variation of inflows from the five tributaries and the water exchange with Changjiang River, the water level fluctuates significantly with alternating periods of floods and droughts, resulting in large seasonally variation of water surface area (Hui et al., 2008; Feng et al., 2012). Generally, the wet-season is from April to September and the dry-season is from October to March (Wang and Liang, 2015). During the wet-season, river-lake flow reversal can occur due to the water level increase of Changjiang River (Shankman et al., 2006). PYL can take in the flood from Changjiang River to reduce the flood risks of downstream area and reach a surface area over 4000 km^2^ and an average depth of 8.4 min summer (Wang and Liang, 2015). During the dry-season, PYL is divided into many connected and disconnected sub-lakes and the lake area can shrink to less than 1000 km^2^. The seasonal reverse-flow system contributes to the spectacular fluctuation of the lake area and the complex yearly hydrological regime leads to seasonal dynamics of the lake ecosystem.

We collected 10 samples in wet-season (2–5 August 2017) and 13 samples in dry-season (10–14 January 2017) (Figure 1). During the sampling, the water level measured at the Xingzi hydrometric station (Figure 1) was 16.5 ± 0.1 m and 9.7 ± 0.1 m in wet-season and dry-season, respectively. In general, the water level of PYL reaches the lowest in December or January and the highest water level in July or August. Thus, these samples can help us to understand the responses of lake nutrients and microbial communities to this extreme water level fluctuation within a year. In order to avoid the influences of storms on wet-season sampling, the samples were collected before the storm. There was also no rainfall before and during dry-season sampling. Due to the severe sand excavation in the northern part of the lake in dry-season, this area was not sampled during the dry-season. In each sample site, water samples were collected at the depth of 0.5 m. 200-mL subsample was filtered through a 0.2-μm Polycarbonate Membrane Filter (Whatman, United Kingdom). Filter was then frozen in liquid nitrogen in the field immediately and stored at -80°C in the lab until DNA extraction. Another 500-mL subsample was acid fixed and transported to the laboratory at 4°C for chemical analyses. According to the Clean Water Act Analytical Methods of United States Environmental Protection Agency, total nitrogen (TN) was analyzed using ion chromatography after persulfate oxidation. Total phosphorus (TP) was tested using the ascorbate acid colorimetric method after oxidation. To test nitrate (NO_3_^-^), ammonium (NH_4_^+^), soluble reactive phosphorus (SRP), and dissolved organic carbon (DOC), water samples were filtered through glass fiber filters (Whatman, United Kingdom). Ion chromatography was used to test NO_3_^-^. Indophenol colorimetric method was used to test NH_4_^+^. Ascorbate acid colorimetric method was used to test SRP. DOC was analyzed using a Shimadzu TOC Analyzer (TOC-VCPH, Shimadzu Scientific Instruments, Columbia, MD, United States).

### DNA Extraction, PCR, and Sequencing

Following manufacturer protocols, DNA was extracted from the filter samples using the TIANGEN-DP336 soil DNA Kit (TIANGEN-Biotech, Beijing, China). The V3-V4 regions were amplified using the forward primer 347F-CCTACGGRRBGCASCAGKVRVGAAT and reverse primer 802R-GGACTACNVGGGTWTCTAATCC (GENEWIZ, Inc., South Plainfield, NJ, United States). PCR was performed using the following program: initial denaturation at 94°C for 3 min, 24 cycles of denaturation at 94°C for 30 s followed by annealing at 57°C for 90 s and extension at 72°C for 10 s, and final extension step at 72°C for 10 min. Amplified DNA was purified using the Gel Extraction Kit (Qiagen, Hilden, Germany) and then validated by Agilent 2100 Bioanalyzer (Agilent Technologies, Palo Alto, CA, United States) and quantified by Qubit 2.0 Fluorometer (Invitrogen, Carlsbad, CA, United States). According to manufacturer’s instructions, DNA libraries were loaded on an Illumina MiSeq instrument (Illumina, San Diego, CA, United States).

### Sequence Analysis and Functional Prediction

Raw sequences were processed using the software package QIIME 1.9.1 (Caporaso et al., 2010). The forward and reverse reads were joined and assigned to samples based on barcode. Then the barcode and primer sequence were cut off and the sequences were qualifying filtered. The effective sequences were clustered into operational taxonomic units (OTUs) at a threshold of 97% similarity against the Silva 132 database (Quast et al., 2013). PICRUSt (Phylogenetic Investigation of Communities by Reconstruction of Unobserved States) was used to make functional prediction from the valid 16S rRNA sequences (Langille et al., 2013). The reference sequences used for PICRUSt prediction were clustered into OTUs against the Greengenes 13.5 database (McDonald et al., 2012) at a cutoff of 97% similarity. The nearest sequence taxon index (NSTI) was calculated using PICRUSt to measure the average phylogenetic distance between OTUs and a gene sequence from a fully sequenced genome (Langille et al., 2013). The average NSTI value in this study was 0.11, indicating high accuracy of PICRUSt prediction. The predicted metagenomes were further clustered into Kyoto Encyclopedia of Genes and Genomes (KEGG) Orthologs (KOs). The KOs associated with carbon and nitrogen metabolisms were identified based on the KEGG database (Kanehisa and Goto, 2000). The KOs associated with phosphorus cycle were identified and grouped into six functional categories (Bergkemper et al., 2016). The Raw sequence data was available at the National Center for Biotechnology Information (PRJNA436872 and SRP133903).

### Statistical Analysis

We compared alpha diversity and taxonomic, functional, and chemical differences between dry-season and wet-season using *t*-test in SPSS 22.0 (IBM, Armonk, NY, United States). Linear discriminant analysis effect size (LEfSe) method (Segata et al., 2011) was performed in QIIME (Caporaso et al., 2010) to identify the specialized bacterial taxa enriched in dry-season and wet-season, respectively. The principal coordinates analysis (PCoA) was performed to reveal seasonal differences of bacterial communities based on Bray–Curtis distances in terms of the relative abundance of OTUs, as well as the relative abundance of overall KOs and KOs associated with C-metabolism, N-metabolism, and P-cycle using the Vegan package 2.4–3 (Oksanen et al., 2007) in R 3.4.4 (R Core Team, 2017). Heatmap showed the distribution of top 100 OTUs in dry-season and wet-season. Analysis of variance using distance matrices (ADONIS, “adonis” function), analysis of similarity (ANOSIM, “anosim” function), and multi-response permutation procedure analysis (MRPP, “mrpp” function) were conducted to test the functional differences (overall function, carbon metabolism, nitrogen metabolism, and phosphorus cycle) between bacterial communities in dry-season and wet-season. Mantel tests were applied to assess the relationships between bacterial community structures (taxonomic and functional) and environmental variables. We performed distance-based redundancy analysis (dbRDA) to reveal the association of the microbial communities with nutrient factors. The goodness of fit for each nutrient factor was estimated by applying the envfit function with 999 permutations. Variance partitioning analysis (VPA) was performed to determine the relative contributions of C-factor (including DOC), N-factor (including TN, NO_3_^-^, and NH_4_^+^), P-factor (including TP and SRP), and the interactions between two or three of these factors (C × N, C × P, N × P, and C × N × P). Spearman correlation analyses (*P*-values were adjusted using FDR method) were performed to assess the relationships between relative abundances of dominant phyla and environmental variables in dry-season and wet-season, respectively.

## Results

### Dynamics of Nutrient Factors

**FIGURE 2:**
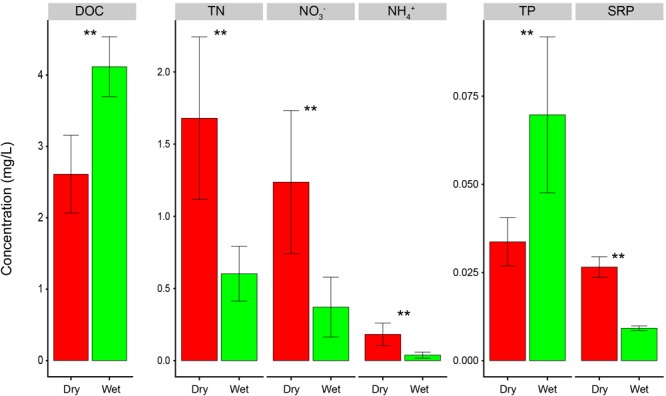
Nutrient concentrations in dry and wet seasons of Poyang Lake, China. Statistical significance between dry and wet seasons was assessed by *t*-test and indicated by asterisks (^∗∗^represents *P* < 0.01).

Poyang Lake had significantly higher concentrations of TN, NO_3_^-^, NH_4_^+^, and SRP in dry-season than in wet-season (*t*-test, *P* < 0.01, Figure 2). DOC and TP were higher in wet-season than in dry-season (*t*-test, *P* < 0.01, Figure 2). In dry-season, NO_3_^-^ and NH_4_^+^ accounted for 72 and 12% of TN, and SRP accounted for 81% of TP on average. In wet-season, NO_3_^-^ and NH_4_^+^ accounted for 62 and 7% of TN, and SRP accounted for only 15% of TP on average. Overall, dry-season had higher environmental dissimilarity than wet-season (Supplementary Figure S1a).

### Dynamics of Taxonomic Composition

**FIGURE 3:**
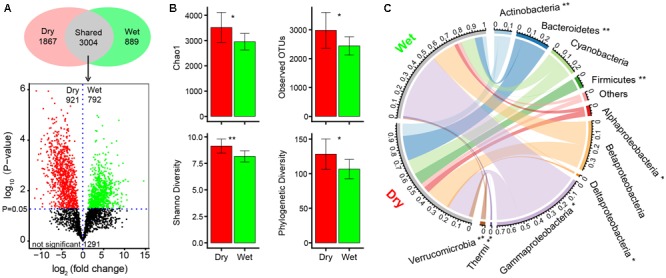
Basic differences of bacterial communities between dry-season and wet-season. **(A)** Venn diagram showing the unique and shared OTUs in dry-season and wet-season. The volcano plot showing the shared OTUs that significantly (*t*-test, *P* < 0.05) enriched in dry-season (red dots) and wet-season (green dots). The volcano plot was constructed using log2 fold change on *x*-axis and –log10 *p*-values of *t*-test on *y*-axis. **(B)** Alpha diversity (Chao 1, observed OTUs, Shannon diversity, and phylogenetic diversity) of bacterial communities in dry-season and wet-season. **(C)** Chord diagram showing the relative abundances of dominant phyla (the phyla with a relative abundance >1%) in dry-season and wet-season. “Others” represents the unsigned OTUs and the phyla with a relative abundance <1%. Statistical significance between dry-season and wet-season was assessed by *t*-test and indicated by asterisks (^∗^represents *P* < 0.05, ^∗∗^represents *P* < 0.01).

The sequencing of 16S rRNA genes yielded 1,247,134 high-quality sequences which were clustered into 5760 OTUs at 97% nucleotide similarity level. In total, 4871 OTUs were detected in dry-season and 3893 in wet-season (Figure 3A and Supplementary Figure S2). Venn diagram shown that 3004 OTUs were shared by dry-season and wet-season and within which, 921 and 792 OTUs had a significantly higher relative abundance in dry-season and wet-season, respectively (*P* < 0.05, Figure 3A). Moreover, 1867 OTUs were unique in dry-season and 889 OTUs were unique in wet-season (Figure 3A and Supplementary Figure S2). In dry season, there were 4 dominant OTUs (relative abundance >1%) belonging to the orders Bacillales, Pseudomonadales, and Cryptophyta (Supplementary Table S1). In wet-season, there were 11 dominant OTUs (relative abundance >1%) belonging to the orders Pseudomonadales, Chroococcales, Stramenopiles, Burkholderiales, Flavobacteriales, and Neisseriales (Supplementary Table S1). Alpha diversity was significantly higher in dry-season than in wet-season (*P* < 0.05, Figure 3B). The average number of observed OTUs were 2980 and 2444 in dry-season and wet-season, respectively.

In dry-season, the dominant phyla (relative abundance >1%) were Proteobacteria, followed by Bacteroidetes, Cyanobacteria, Actinobacteria, Firmicutes, and Verrucomicrobia (Figure 3C). In wet-season, the dominant phyla (relative abundance >1%) were Proteobacteria, followed by Cyanobacteria, Bacteroidetes, Actinobacteria, and Thermi (Figure 3C). Comparing relative abundances of these dominant phyla between dry-season and wet-season, Actinobacteria, Alphaproteobacteria, Bacteroidetes, Deltaproteobacteria, Firmicutes, and Verrucomicrobia had a significantly higher relative abundance, while Gammaproteobacteria and Thermi had a significantly lower relative abundance in dry-season than in wet-season (Figure 3C). The LEfSe method identified a suite of specialized bacterial taxa enriched in dry-season and wet-season, respectively (Supplementary Figure S3). Actinobacteria, Bacteroidetes, Firmicutes, Verrucomicrobia, as well as the classes of Chloroplast, Alpha- and Delta-proteobacteria were notably discriminated as dominant key groups in dry-season. Proteobacteria (particularly Gammaproteobacteria) and the classes of Cyanobacteria including Synechococcophycideae, Oscillatoriophycideae, and Nostocophycideae were discriminated in wet-season.

**FIGURE 4:**
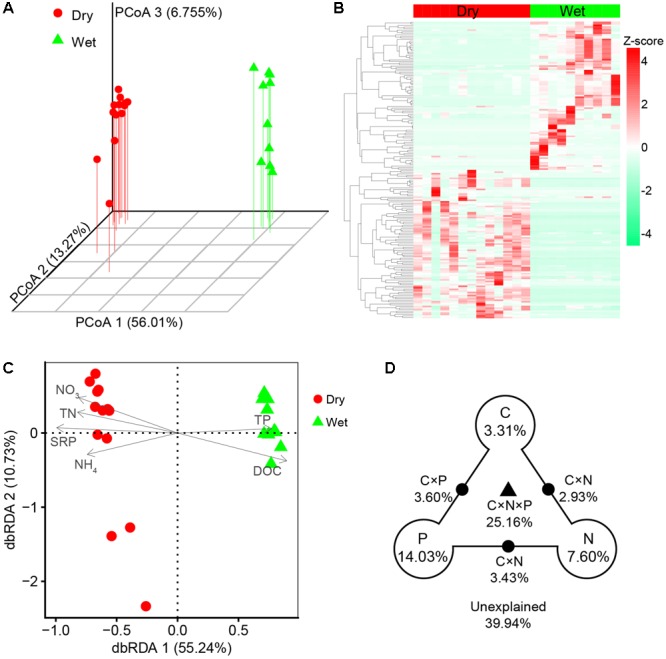
**(A)** Principal coordinates analysis (PCoA) based on Bray–Curtis distance in terms of the relative abundance of OTUs. **(B)** Z-score normalized heatmap of the top 100 OTUs. **(C)** Distance-based redundancy analysis (dbRDA) of bacterial community (symbols) and nutrient variables (arrows). All nutrient variables had goodness of fit at the significant level *P* < 0.05 by envfit function. **(D)** Variance partition analysis (VPA) determined the relative contributions of C-factor (including DOC), N-factor (including TN, NO_3_^-^, and NH_4_^+^), P-factor (including TP and SRP), and the interactions between two or three of these factors (C × N, C × P, N × P, and C × N × P). The relative variance proportions that the corresponding components could explain are shown in percentages. Blank circles on the end of the triangle show the percentage of variance explained by C, N, and P alone. The percentage of variance explained by interactions between two or three of these factors is depicted as black dots on the edges or black triangle in the middle.

**FIGURE 5:**
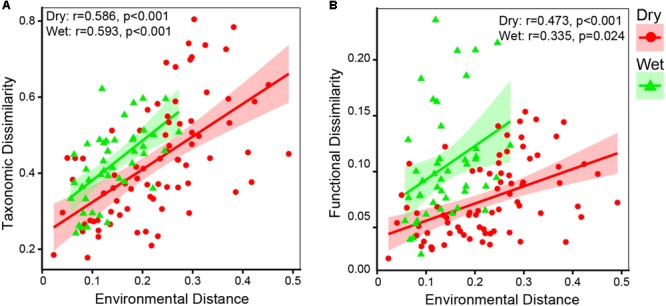
Results of Mantel test **(A)** between taxonomic matrix and environmental matrix, and **(B)** between functional matrix and environmental matrix. Spearman correlation coefficients (r) and associated *p*-values were calculated. Taxonomic dissimilarity and functional dissimilarity were represented by Bray–Curtis distances in terms of relative abundance of OTUs and KOs, respectively. Environmental distance is represented by Euclidean distance in terms of environmental variables (DOC, TN, NO_3_^-^, NH_4_^+^, TP, and SRP).

Principal coordinates analysis and heatmap revealed that bacterial communities were distinct between dry-season and wet-season (Figure 4A,B). The bacterial communities in different seasons were mainly separated along the first axis (PCoA 1, 56.01%, Figure 4A). Bacterial communities in dry-season and wet-season were not significantly different in taxonomic beta-diversity (Supplementary Figure S1b). However, taxa in dry-season exhibited greater niche width values than taxa in wet-season (Supplementary Figure S1c). Mantel tests revealed that taxonomic dissimilarities (Bray–Curtis distance based on the relative abundance of OTUs) showed positive correlations with environmental distance in both dry- and wet-season (Figure 5A). dbRDA indicated that the spatiotemporal variation of bacterial communities significantly associated with changes in nutrient factors including DOC, TN, NO_3_^-^, NH_4_^+^, TP, and SRP (*P* < 0.05, Figure 4C). As indicated by VPA, nutrient factors explained 60.06% of the composition variance of these bacterial communities, which contributed by C-factor (3.31%), N-factor (7.60%), P-factor (14.03%), and interactions of two or three of these factors (Figure 4D). Mantel tests revealed that taxonomic structures of bacterial community were closely correlated with variations of DOC, TN, NO_3_^-^, and SRP in dry-season, and with variations of NO_3_^-^ and TP in wet-season (Supplementary Table S2). More specifically, the relative abundances of Bacteroidetes, Cyanobacteria, and Verrucomicrobia were positively correlated with NO_3_^-^ and SRP while negatively correlated with DOC in dry-season (Supplementary Figure S4). Firmicutes and Gammaproteobacteria were negatively correlated with NO_3_^-^ and SRP but positively correlated with DOC in dry-season (Supplementary Figure S4). However, the relative abundances of dominant phyla were not significantly correlated with nutrient concentrations in wet-season (Supplementary Figure S4).

### Dynamics of Functional Composition

**FIGURE 6:**
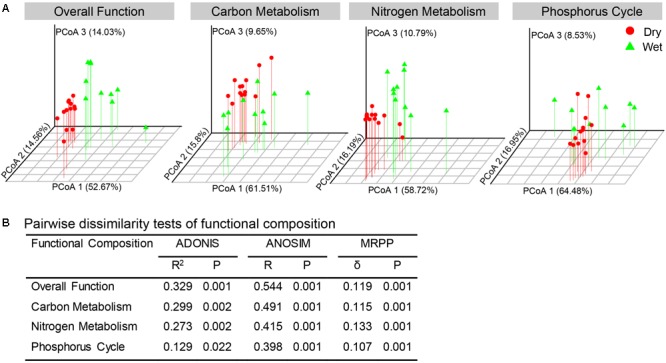
Distribution and distinction of functional composition of the bacterial communities between dry-season and wet-season. **(A)** PCoA based on Bray–Curtis distance in terms of relative abundances of KOs associated to overall function, C-metabolism, N-metabolism, and P-cycle. **(B)** Non-parametric statistical tests of ADONIS (analysis of variance using distance matrices), ANOSIM (analysis of similarity), and MRPP (multi-response permutation procedure analysis).

Based on the PICRUSt metagenome prediction, 6237 KOs were predicted from 16S rRNA gene sequences. PCoA and non-parametric statistical tests (adonis, ANOSIM, and MRPP) revealed that bacterial communities in dry-season and wet-season had distinct functional compositions (Figure 6). The functional compositions of bacterial communities in different seasons were mainly separated along the first axis (Figure 6A). Wet-season had significantly higher functional beta-diversity than dry-season (Supplementary Figure S1d).

**FIGURE 7:**
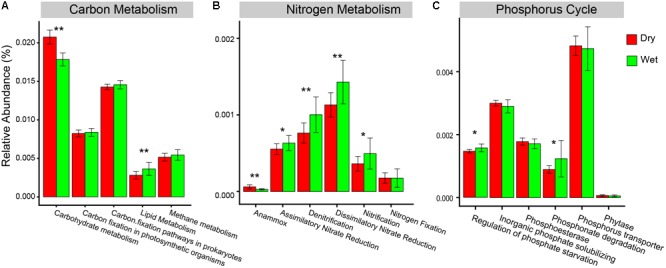
Relative abundances of the major KEGG pathways associated with **(A)** carbon metabolism, **(B)** nitrogen metabolism, and **(C)** functional categories of phosphorus cycle. Statistical significance between dry-season and wet-season was assessed by *t*-test and indicated by asterisks (^∗^represents *P* < 0.05, ^∗∗^represents *P* < 0.01).

For the predicted functions in the pathways of C-metabolism, N-metabolism, and P-cycle, carbohydrate metabolism and anammox had a significantly higher relative abundance in dry-season than in wet-season (Figure 7). However, lipid metabolism, assimilatory nitrate reduction, denitrification, dissimilatory nitrate reduction, nitrification, phosphonate degradation, and regulation of phosphate starvation had a significantly higher relative abundance in wet-season than in dry-season (Figure 7).

**FIGURE 8:**
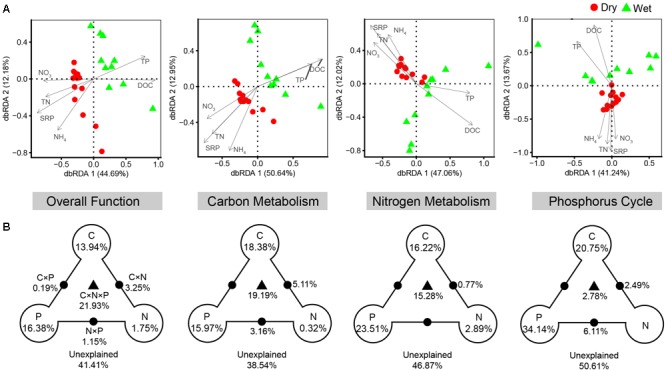
Relationship between the functional composition and nutrient variables. **(A)** Distance-based redundancy analysis (dbRDA) of the functional composition of the bacterial communities (symbols) and nutrient variables (arrows), in terms of overall function, carbon metabolism, nitrogen metabolism, and phosphorus cycle. **(B)** Corresponding variance partition analysis (VPA) determined the relative contributions of carbon (C, including DOC), nitrogen (N, including TN, NO_3_^-^, and NH_4_^+^), phosphorus (P, including TP and SRP), and the interactions between two or three of these factors (C × N, C × P, N × P, and C × N × P). The relative variance proportions that the corresponding components could explain are shown in percentages on the right. Blank circles on the end of the triangle show the percentage of variance explained by C, N, and P alone. The percentage of variance explained by interactions between two or three of these factors is depicted as black dots on the edges or black triangle in the middle.

Consistent with taxonomic dissimilarity, functional dissimilarity also showed positive correlations with environmental distance in both dry- and wet-season (Figure 5B). The results of dbRDA indicated that spatiotemporal variations of functional composition (overall function, C-metabolism, N-metabolism, P-cycle) were significantly associated with changes in nutrient factors, including DOC, TN, NO_3_^-^, NH_4_^+^, TP, and SRP (*P* < 0.05, Figure 8). VPA indicated that nutrient factors explained 58.59, 61.46, 53.13, and 49.39% of the functional variances in terms of overall function, C-metabolism, N-metabolism, and P-cycle, respectively (Figure 8). P-factor, C-factor, and the interaction of C, N, and P factors (C × N × P) contributed the most to the functional variances. Mantel tests further demonstrated that functional dissimilarities were closely correlated with variations of DOC, NO_3_^-^, and SRP in dry-season, while with variations of DOC and TP in wet-season (Supplementary Table S2).

## Discussion

In PYL, the nutrient concentrations showed dramatic seasonal patterns. The results suggest that PYL was N enriched in dry-season while DOC and P enriched in wet-season. In wet-season, stormwater carry a large amount of suspended solids into the lake and often has a high loading of TP, within which, the largest component is particulate phosphorus (Massey and Jackson, 1952; Kronvang et al., 1997; Duan et al., 2012; River and Richardson, 2018). In shallow lakes with great WLFs, the changes of water quality during a hydrological year were significantly controlled by WLFs (Wantzen et al., 2008; White et al., 2008; Stefanidis and Papastergiadou, 2013). In general, water quality responds immediately to WLFs and was worse with high nutrient concentrations in dry-season than in wet-season, because in dry-season, the lake have a low water capacity for dilution and degradation, low biological consuming of nutrients, and high release of nutrients from sediment to water (Zhu et al., 2013; Yao et al., 2015; Li et al., 2016; Liu X. et al., 2016; Hideo et al., 2017). For PYL particularly, previous studies about the water quality variation associated to WLFs demonstrated high nitrogen concentrations in dry-season (Wu et al., 2006; Yao et al., 2015; Liu X. et al., 2016), compositional changes in DOC related to hydrological regime (White et al., 2008; Yao et al., 2015), and complex distribution patterns of phosphorus (Xiang and Zhou, 2011; Wang and Liang, 2015). Considering the responses of bacterial communities to environmental variations, these seasonal nutrient dynamics in PYL are important drivers of dynamic patterns of bacterial communities in dry-season and wet-season.

Our results showed that bacterial communities were distinct taxonomically and functionally in dry-season and wet-season in PYL, suggesting great impacts of seasonal WLF on lake bacterial communities. Bacterial communities had a higher alpha diversity in dry-season than in wet-season. Large seasonal variabilities in bacterial community compositions were also detected. In dry-season, Actinobacteria, Bacteroidetes, Firmicutes, Verrucomicrobia, as well as Alpha- and Delta-proteobacteria were discriminated as the dominant key groups. Some of these groups are known to perform nucleic and amino acid metabolism and degradation of organic compounds (Newton et al., 2011). High NO_3_^-^ and SRP concentrations in dry-season could stimulate the growth of Bacteroidetes, Cyanobacteria, and Verrucomicrobia while inhibit Firmicutes and Gammaproteobacteria. In wet-season, however, only Gammaproteobacteria and some class of Cyanobacteria were discriminated as dominant key groups, which are known to perform photosynthesis and nitrogen fixation (Newton et al., 2011). The relative abundances of dominant phyla were not significantly correlated with nutrient concentrations in wet-season. Moreover, some microorganisms can come from adjacent terrestrial ecosystems (Bardgett et al., 2007; Schuette et al., 2010). The dispersal of soil bacteria can be an important driver of microbial community structure in aquatic ecosystems (Nepf, 2012; Taherzadeh et al., 2012). Thus, the variations of water-land interface caused by WLFs could influence the dispersal of soil bacteria to PYL, influencing microbial communities in the lake. Many previous studies focused their interests on the effect of WLFs on periphyton, macrophytes, macroinvertebrates, and fish (Coops et al., 2003; Baumgaertner et al., 2008; Brauns et al., 2008; Sutela and Vehanen, 2008; White et al., 2008). In general, WLFs shift the community structures and lead to diversity decreases of these biota due to the changes of hydrological regime (particularly timing and amplitudes) and habitat (particularly habitat loss) (Riis and Hawes, 2002; Turner et al., 2005; Brauns et al., 2008; Sutela and Vehanen, 2008). Different to these biota, bacterial communities had a higher alpha-diversity in dry-season. In addition, our study also demonstrated a high contribution of nutrient variations to the spatiotemporal variation of bacterial communities, especially P, N, and the interactions of C, N, and P (C × N × P), suggesting strong influences of nutrients on bacterial community differences between dry-season and wet-season. Furthermore, bacterial communities respond differently to nutrient variations in dry-season and wet-season. In dry-season, DOC, TN, NO_3_^-^, and SRP were main drivers of bacterial taxonomic variation, while in wet-season, the main drivers were NO_3_^-^ and TP, suggesting that bacterial communities in dry-season were more intimately correlated with nutrient variations. The results consistent with other studies (Judd et al., 2006; Peter et al., 2011) by indicating that nutrients were potential drivers of microbial communities in PYL, especially in dry-season.

In wet-season, most of the metabolism pathways associated with N-metabolism had higher relative abundance of genes than in dry-season, including assimilatory nitrate reduction, denitrification, dissimilatory nitrate reduction, and nitrification. Wet-season is the growing season when the microbial growth synchronizes with high nutrient absorption and utilization abilities, leading to high requirements of N-associated enzymes and suggesting that bacterial communities are potentially more efficient at removing nitrogen in wet-season. Moreover, PYL is one of the lakes facing serious threat of eutrophication in China (Wang et al., 2015; Zhang et al., 2015; Liu J. et al., 2016). The intense agricultural activities in the catchment of PYL aggravated nutrients loading to the lake (Wang and Liang, 2015; Liu J. et al., 2016). PYL had high nitrogen concentrations all year round with an even higher concentration in dry-season. Denitrification and anammox are two important metabolic pathways to remove nitrogen from aquatic ecosystems (Tiedje et al., 1983; Seitzinger, 1988). In PYL, bacterial communities had higher relative abundance of genes associated to denitrification in wet-season than in dry-season. However, anammox had higher relative abundance of associated genes in dry-season than in wet-season. These results suggest different nitrogen removal mechanisms in different seasons. In wet-season, the bacterial communities also had higher relative abundance of genes associated to P-cycle (phosphonate degradation and regulation of phosphate starvation) and this was consistent with high TP but low SRP concentrations in wet-season. The functional traits in N-metabolism and P-cycle are valuable ecological markers to understand the roles of microbial communities in biogeochemical processes and to reveal their responses to environmental changes (Green et al., 2008; Barberan et al., 2012; Fierer et al., 2012; Freedman and Zak, 2015; Ren et al., 2017). In different seasons, the functional composition of bacterial communities responded differently to nutrient dynamic. Similar to the responses of taxonomic composition, DOC, NO_3_^-^, and SRP were main drivers of functional dissimilarities in dry-season, while TP was the main driver in wet-season.

Through evaluation of the dynamics of taxonomic and functional compositions in dry-season and wet-season, our study suggested that the nutrient contributed more strongly to the variations of bacterial communities in dry-season than in wet-season. We attributed the lower contribution of nutrient variation in wet-season to the complex hydrological characteristics. In wet-season, PYL has the highest water level but the lowest velocities due to the large amount of inputs from its five tributaries as well as the backflows of the Changjiang River (Li et al., 2016). Compared to its catchment inflow, the Changjiang River discharge has a greater impact on intra-annual WLFs of PYL (Ye et al., 2014). The strong impacts of Yangtze River in wet-season drive the lake homogenization, which reflected on the strongly clustered bacterial communities and lower nutrient contributions to bacterial community variation in wet-season.

Our study revealed the spatiotemporal patterns of bacterial community distributions as well as their functional potentials during an extreme seasonal WLF with concomitant change of nutrients. However, more in-depth work is required to overcome some of our study’s limitations and to gain profound understanding of WLFs consequences. For example, this study was conducted only in January and August to represent dry-season and wet-season, respectively. A year-round and long term inter-annual investigations can help to build a broader knowledge of the impacts caused by both inter- and intra-annual WLFs. Moreover, in addition to nutrients, more environmental variables should be considered, such as pH, temperature, hydrology, heavy metals, and organic pollutions, because all these factors can potentially contribute to microbial community variations.

## Conclusion

Water level fluctuations (WLFs) are a pervasive pressure on lacustrine ecosystems globally. In lake ecosystems, various organisms are affected by WLFs. In this study, we revealed the dynamic patterns of bacterial communities taxonomically and functionally during a huge intra-annual WLF in a large shallow lake. The nutrient concentrations showed dramatic seasonal patterns with higher N in dry-season, while higher DOC and P in wet-season. Both taxonomic and functional compositions of the bacterial communities were distinct in dry-season and wet-season. Nutrients play as important drivers of the seasonal variation of bacterial communities. However, bacterial communities responded differently to nutrient dynamics in different seasons. The bacterial communities are taxonomically sensitive in dry-season while more functionally sensitive in wet-season. Bacterial communities are more efficient in nutrients utilization in wet season and had different nitrogen removal mechanisms in different seasons. Bacterial communities in wet-season had significantly higher relative abundance of denitrification genes but lower anammox genes than in dry-season. The results can enrich our understanding of seasonal dynamics of lake ecosystems and improve our abilities to predict impacts of WLFs, providing important implications for the conservation and management of lake ecosystems globally in respond to the increasing pressures of climate change and anthropogenic activities.

## Data Availability

The datasets generated for this study can be found in National Center for Biotechnology Information, PRJNA436872 and SRP133903.

## Author Contributions

ZR and XQ designed the study, did the analyses, and prepared the manuscript. XQ, MZ, and YY performed the field work and laboratory work. WP provided suggestions during the entirety of the study.

## Conflict of Interest Statement

The authors declare that the research was conducted in the absence of any commercial or financial relationships that could be construed as a potential conflict of interest.
