# Supplementary material for: Distinct Bacterial Communities in Wet and Dry Seasons During a Seasonal Water Level Fluctuation in the Largest Freshwater Lake (Poyang Lake) in China
Source: Front Microbiol. 2019 May 21;10:1167. doi: 10.3389/fmicb.2019.01167 (PMC6536640; doi:10.3389/fmicb.2019.01167)
Supplement: Supplementary file 1 [file Data_Sheet_1.docx]

***Supplementary Material***

Figure S 1 Comparisons of dry-season versus wet-season in (a) taxonomic dissimilarity, (c) average taxa niche width, and (b) functional dissimilarity of the bacterial communities, as well as (d) environmental dissimilarity. Statistical significance between dry and wet seasons was assessed by t-test and indicated by asterisks (∗∗ represents P < 0.01).


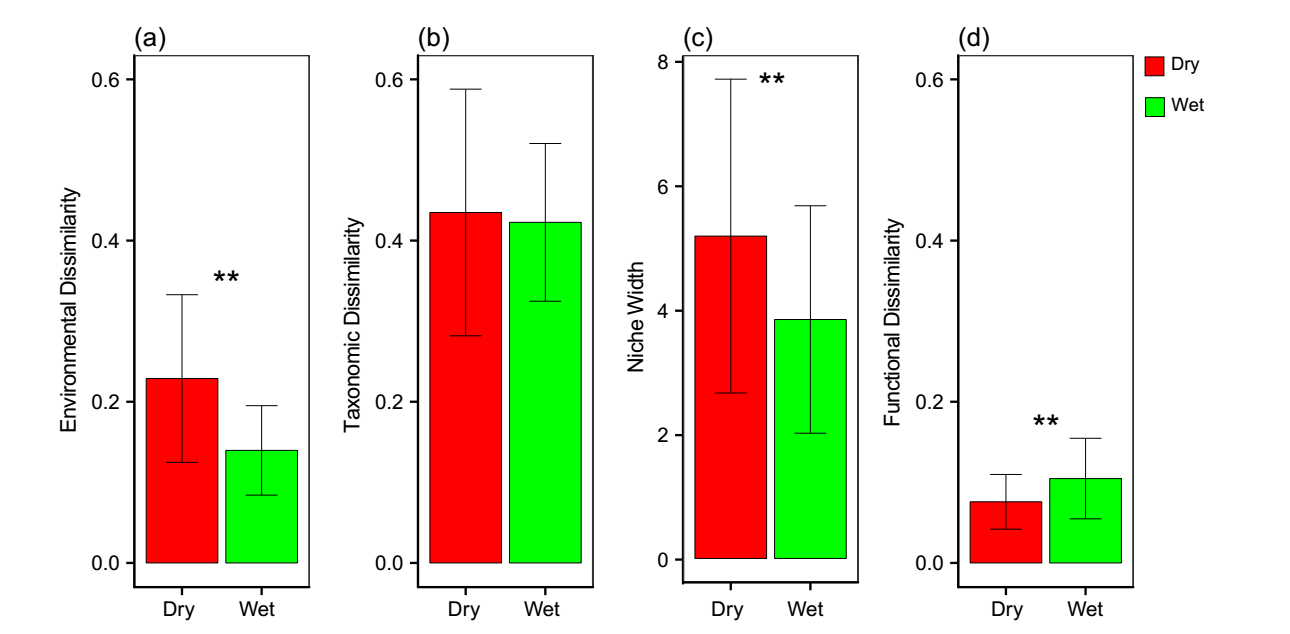


Figure S 2 Pie charts shown the proportion of OTUs belonging to dominant phyla in dry-season and wet-season, respectively in terms of (a) all OTUs and (b) seasonally unique OTUs.


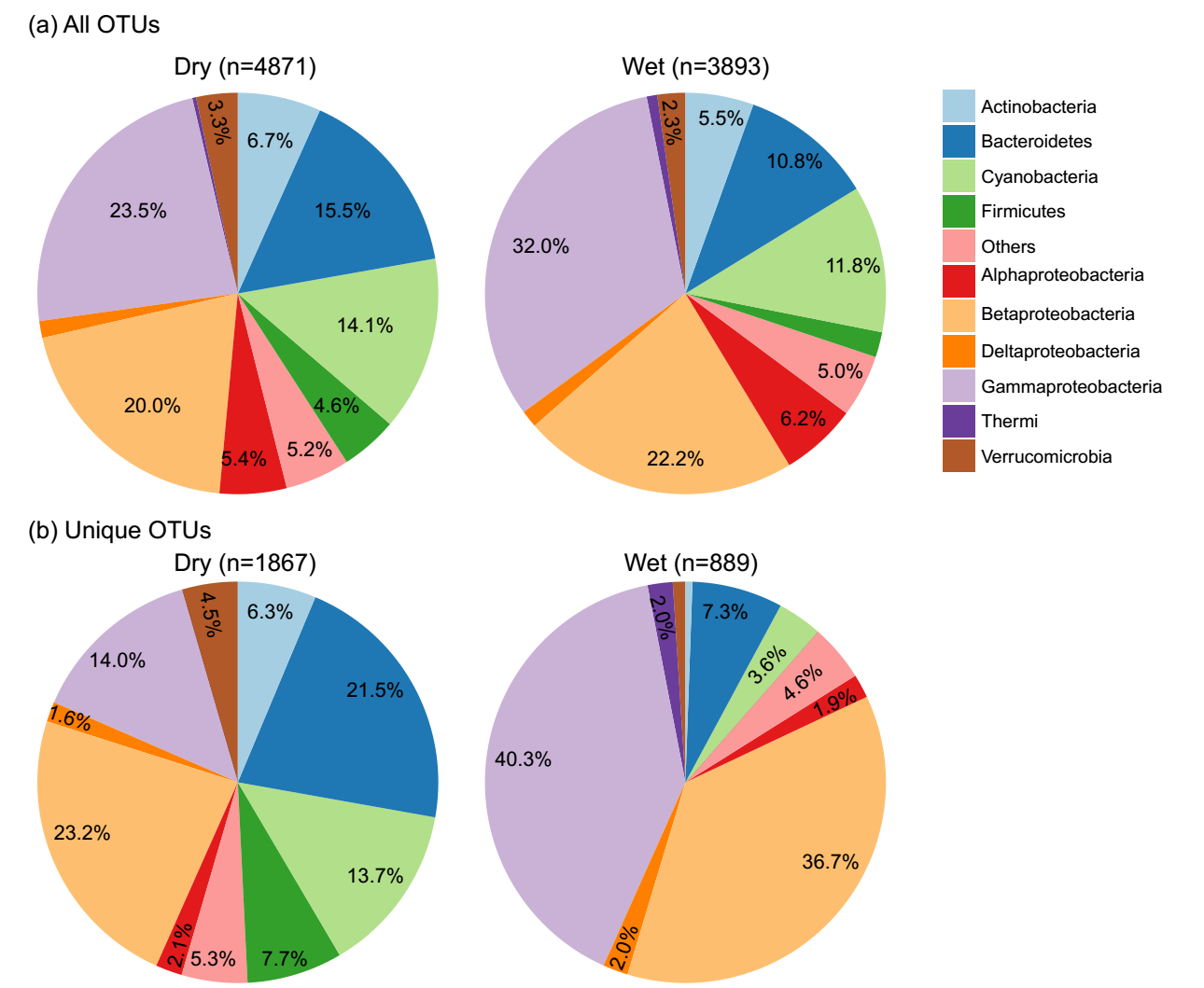


Figure S 3 LEfSe (Linear discriminant analysis effect size, LDA >2.0) taxonomic cladogram classifies criminative taxonomic differences between dry (red) and wet (green) seasons’ bacterial communities from phylum to genes level (relative abundance ≥0.01 %) based on 16s rRNA gene sequences. Significantly discriminant taxon nodes are colored, and branch areas are shaded according to the highest ranked group for that taxon. Taxa with non-significant differences are represented as brown symbols.


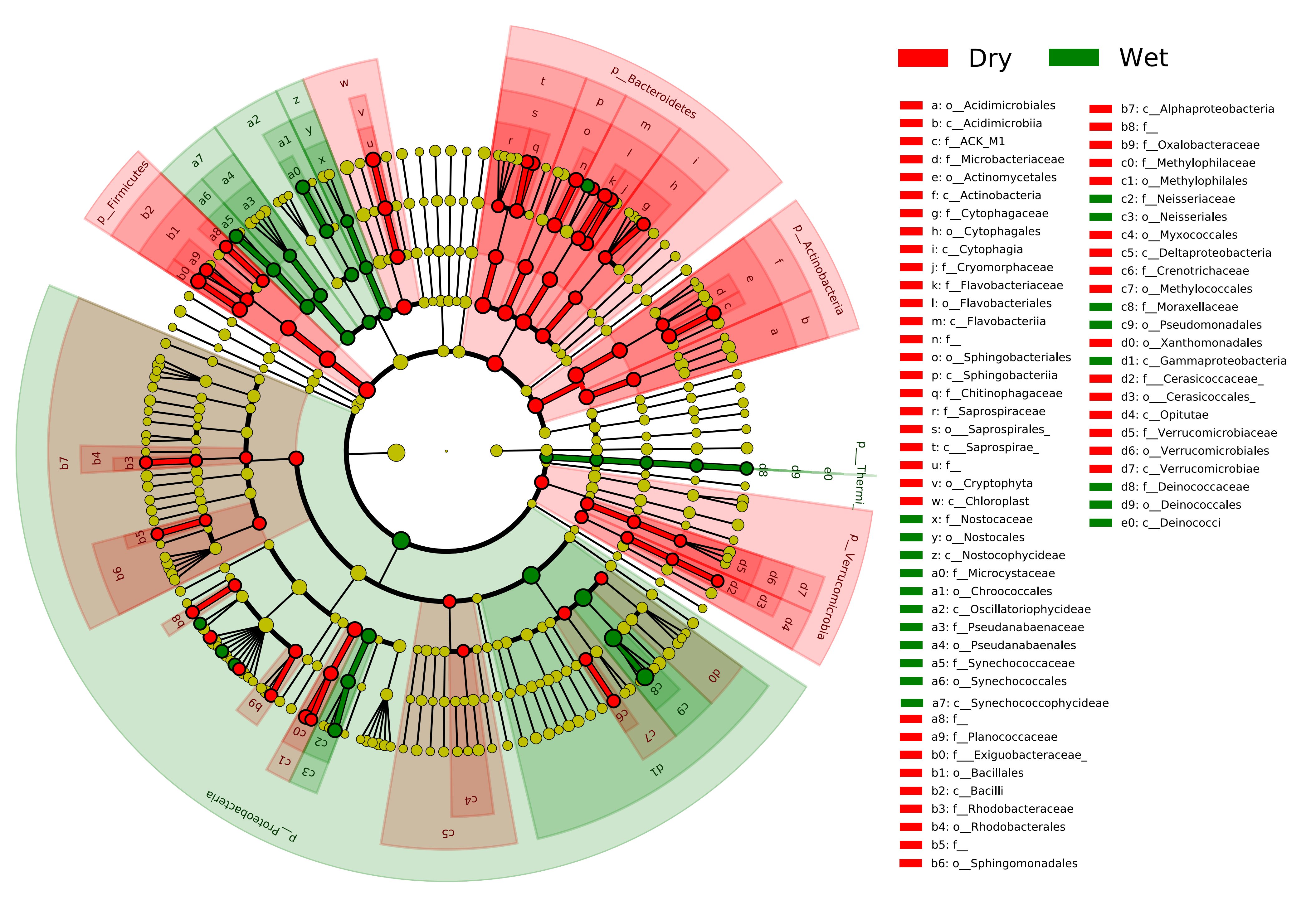


Figure S 4 Spearman correlations between the relative abundance of dominant phyla and environment variables in dry-season and wet-season. Green color represents negative correlation. Red color represents positive correlation. P-values were adjusted using FDR method.


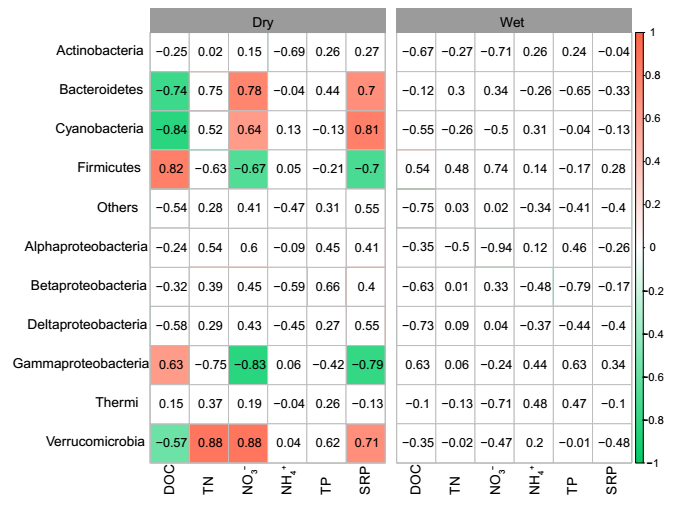


Table S 1 The most abundant OTUs and their relative abundances in dry-season and wet-season. (OTUs with a relative abundance >0.01)

| #OTU ID | Dry | Wet | Taxonomy |
| --- | --- | --- | --- |
| denovo3130 | 0.024 | 0.000 | p_Firmicutes; c_Bacilli; o_Bacillales; f_[Exiguobacteraceae]; g_Exiguobacterium; s_ |
| denovo62050 | 0.023 | 0.000 | p_Proteobacteria; c_Gammaproteobacteria; o_Pseudomonadales; f_Moraxellaceae; g_Acinetobacter; s_ |
| denovo72728 | 0.011 | 0.000 | p_Cyanobacteria; c_Chloroplast; o_Cryptophyta; f_; g_; s_ |
| denovo83901 | 0.010 | 0.000 | p_Firmicutes; c_Bacilli; o_Bacillales; f_Planococcaceae; g_Paenisporosarcina; s_ |
| denovo43220 | 0.004 | 0.053 | p_Proteobacteria; c_Gammaproteobacteria; o_Pseudomonadales; f_Moraxellaceae; g_Acinetobacter; s_ |
| denovo51275 | 0.005 | 0.049 | p_Proteobacteria; c_Gammaproteobacteria; o_Pseudomonadales; f_Moraxellaceae; g_Acinetobacter; s_rhizosphaerae |
| denovo8438 | 0.000 | 0.026 | p_Proteobacteria; c_Gammaproteobacteria; o_Pseudomonadales; f_Moraxellaceae; g_Acinetobacter; s_ |
| denovo70518 | 0.004 | 0.023 | p_Proteobacteria; c_Gammaproteobacteria; o_Pseudomonadales; f_Moraxellaceae; g_Acinetobacter; s_ |
| denovo105244 | 0.000 | 0.022 | p_Proteobacteria; c_Gammaproteobacteria; o_Pseudomonadales; f_Moraxellaceae; g_Acinetobacter; s_ |
| denovo49053 | 0.000 | 0.021 | p_Cyanobacteria; c_Oscillatoriophycideae; o_Chroococcales; f_Microcystaceae; g_Microcystis; s_ |
| denovo7159 | 0.005 | 0.019 | p_Cyanobacteria; c_Chloroplast; o_Stramenopiles; f_; g_; s_ |
| denovo57128 | 0.000 | 0.019 | p_Proteobacteria; c_Betaproteobacteria; o_Burkholderiales; f_Comamonadaceae; g_; s_ |
| denovo107447 | 0.000 | 0.015 | p_Proteobacteria; c_Gammaproteobacteria; o_Pseudomonadales; f_Moraxellaceae; g_Acinetobacter; s_ |
| denovo97933 | 0.000 | 0.013 | p_Bacteroidetes; c_Flavobacteriia; o_Flavobacteriales; f_[Weeksellaceae]; g_Cloacibacterium; s_ |
| denovo14010 | 0.000 | 0.011 | p_Proteobacteria; c_Betaproteobacteria; o_Neisseriales; f_Neisseriaceae; g_Vogesella; s_ |

Table S 2 Correlation analysis between microbial community structures (overall OTUs, overall KOs, KOs associated with C-metabolism, N-metabolism, and P-cycle) and environmental variables. Spearman correlations were calculated by Mantel test. Significant correlation coefficients (p<0.05) are shown in bold. ∗ represents P < 0.05, ∗∗ represents P < 0.01.

| Environmental  variables | Seasons | Overall  Taxa | |  | Overall  Functions | |  | Carbon  Metabolism | |  | Nitrogen  Metabolism | |  | Phosphorus  Cycle | |
| --- | --- | --- | --- | --- | --- | --- | --- | --- | --- | --- | --- | --- | --- | --- | --- |
| DOC | Dry | **0.850**** |  |  | **0.906**** |  |  | **0.846**** |  |  | **0.807**** |  |  | **0.745**** |  |
|  | Wet | 0.292 |  |  | **0.307*** |  |  | **0.322*** |  |  | 0.254 |  |  | 0.286 |  |
| TN | Dry | **0.303**** |  |  | 0.151 |  |  | 0.060 |  |  | 0.144 |  |  | 0.122 |  |
|  | Wet | -0.166 |  |  | -0.190 |  |  | -0.191 |  |  | -0.196 |  |  | -0.186 |  |
| NO_3_^-^ | Dry | **0.715**** |  |  | **0.649**** |  |  | **0.558**** |  |  | **0.599**** |  |  | **0.530**** |  |
|  | Wet | **0.337*** |  |  | 0.170 |  |  | 0.163 |  |  | 0.157 |  |  | 0.131 |  |
| NH_4_^+^ | Dry | 0.138 |  |  | 0.051 |  |  | 0.123 |  |  | 0.065 |  |  | 0.127 |  |
|  | Wet | -0.013 |  |  | -0.038 |  |  | -0.005 |  |  | 0.026 |  |  | 0.049 |  |
| TP | Dry | -0.037 |  |  | -0.013 |  |  | -0.009 |  |  | -0.050 |  |  | 0.042 |  |
|  | Wet | **0.545**** |  |  | 0.282 |  |  | **0.305*** |  |  | **0.317*** |  |  | **0.318*** |  |
| SRP | Dry | **0.694**** |  |  | **0.708**** |  |  | **0.632**** |  |  | **0.660**** |  |  | **0.577**** |  |
|  | Wet | -0.038 |  |  | 0.016 |  |  | -0.007 |  |  | 0.018 |  |  | -0.012 |  |
